# Correlation between Trunk Posture and Neck Reposition Sense among Subjects with Forward Head Neck Postures

**DOI:** 10.1155/2015/689610

**Published:** 2015-10-25

**Authors:** Han Suk Lee, Hyung Kuk Chung, Sun Wook Park

**Affiliations:** ^1^Department of Physical Therapy, Faculty of Health Science, Eulji University, 212 Yangji-dong, Sujeong-gu, Seongnam, Gyeonggi-do 461-713, Republic of Korea; ^2^Department of Physical Therapy, Ansan University, 155 Ansan University Road, Ansan, Gyeonggi-do 426-701, Republic of Korea; ^3^Department of Physical Therapy, Samsung Seoul Hospital, 81 Irwon Ro, Gang Nam-gu, Seoul 135-710, Republic of Korea

## Abstract

*Objective*. To assess the correlation of abnormal trunk postures and reposition sense of subjects with forward head neck posture (FHP). *Methods*. In all, postures of 41 subjects were evaluated and the FHP and trunk posture including shoulder, scapular level, pelvic side, and anterior tilting degrees were analyzed. We used the head repositioning accuracy (HRA) test to evaluate neck position senses of neck flexion, neck extension, neck right and left side flexion, and neck right and left rotation and calculated the root mean square error in trials for each subject. Spearman's rank correlation coefficients and regression analysis were used to assess the degree of correlation between the trunk posture and HRA value, and a significance level of *α* = 0.05 was considered. *Results*. There were significant correlations between the HRA value of right side neck flexion and pelvic side tilt angle (*p* < 0.05). If pelvic side tilting angle increases by 1 degree, right side neck flexion increased by 0.76 degrees (*p* = 0.026). However, there were no significant correlations between other neck motions and trunk postures. *Conclusion*. Verifying pelvic postures should be prioritized when movement is limited due to the vitiation of the proprioceptive sense of neck caused by FHP.

## 1. Introduction

Forward head neck posture (FHP) is caused by maintaining an abnormal posture for a long time.

This posture shortens the sternoclenoidomastoid (SCM) and scalenus anterior but lengthens the levator scapulae and semispinalis capitis posterior major [1, 2]. Moreover, this posture accelerates neck extensor activity because of upper cervical excessive extension [3, 4]. The activities of the upper and lower trapezius increase as well with this posture [5].

FHP can produce problems related to the proprioception of muscles [6], such as mechanoreceptor function, and alter the sensitivity of spindles of the neck muscles mentioned previously, as well as inducing the loss of kinesthetic acuity of neck motions [7].

Proprioceptive dysfunction [8, 9], reposition sense [7], dizziness [10], coordination [11], balance [12], or others are also affected. In order to solve these issues, various treatments such as craniocervical flexion training [13], eye-head neck coordination exercise [11, 14, 15], mobilization, manipulation [16–18], and cocontraction exercises [19] have been developed.

However, most of these studies only assessed chronic neck pain patients [7–9, 11, 12], not patients with FHP.

Nagai et al. [9] suggested patients with neck pain might have a greater association with total head excursion rather than FHP. This study found that pilots with neck pain had limited motions of the neck but did not have problems related to postures, including FHP or shoulder posture, indicating that neck pain is not always accompanied by FHP.

Therefore, the proprioception of patients with FHP, not those with neck pain, should be studied. Additionally, most of the studies on improving the neck reposition sense focused on the neck itself.

The upper body posture of FHP subjects, similar to that in thoracic kyphosis [20] or rounder shoulders [21, 22], can easily get affected because the neck muscles are anatomically connected to the trunk. Therefore, if we consider the anatomical orientation of the muscles, there could be problems not only with thoracic kyphosis or rounder shoulder but also with other postures of a trunk such as shoulder or pelvic tilt.

Falla et al. [23] found that FHP associated with prolonged sitting can aggravate neck pain, and a neck pain patient with FHP has reduced ability to maintain an upright posture. Ahn [2] and Murphy et al. [24] suggested that pelvic distortion can cause dysfunctions in the cervical spine, and cervical range of motion improved after the pelvic distortion was corrected. Furthermore, the neck muscle is connected to the trunk muscles with fascia [25]. Therefore, trunk posture should be considered if correction of neck problem is needed.

As mentioned above, a FHP subject possibly has a problem with the repositioning sense of the neck, which can be related with the posture of the trunk owing to the anatomical connection with the neck muscles.

The objective of this study was to identify if accuracy of neck motion is affected by trunk posture and present the baseline data on therapy in the clinic in order to ameliorate repositioning sense of the neck in subjects with FHP.

Therefore, we hypothesized that FHP causes loss of kinesthetic accuracy of neck motions, and this kinesthetic accuracy can be affected by trunk posture, including shoulder tilting angle, scapular level, pelvic side tilting, and anterior tilting angle.

## 2. Material and Method

### 2.1. Subjects

From December 2014 to March 2015, 41 subjects (mean age 23.7 ± 2.7 years, mean height 173.6 ± 6.6 cm, mean weight 68.1 ± 10.1 kg, and mean FHP 6.9 ± 2.6 cm) were recruited in the study. The Eulji University approved the study (Grant number EU 14-61), and all subjects were informed of the purpose of this study and provided their written informed consent prior to their participation. This study adhered to the ethical principles of the Declaration of Helsinki. Inclusion criteria were as follows: FHP above 2.5 cm, age 20 to 30 years, no history of concussion or mild neck injury in the previous 12 months, and no other past neurological disorder or fracture.

### 2.2. Experimental Process

**Figure 1:**
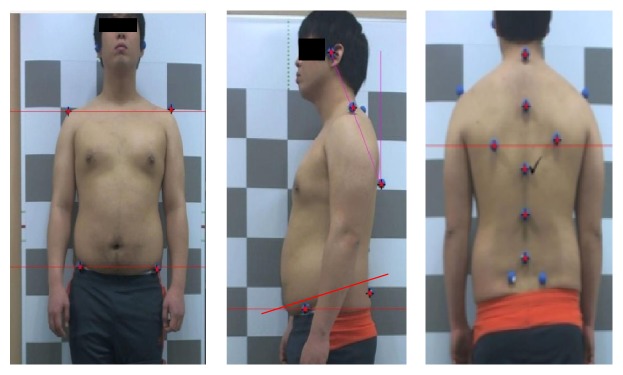
Anterior, lateral, and posterior view.

**Figure 2:**
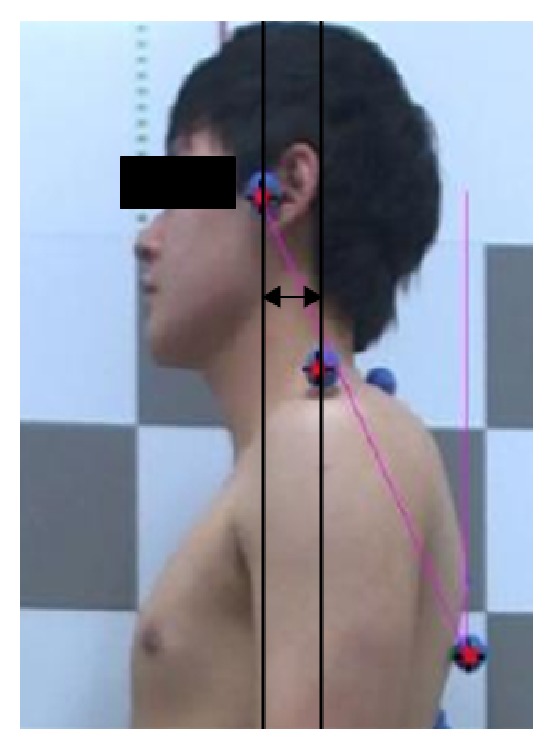
Measurement of FHP.

We measured posture using the Body Style S-8.0 (South Korea, LU Commerce) and used the Body Style Analyzer (System Software) to evaluate the posture. We used body markers over each landmark, including the tragus of the ear, the spinous process of the C7 vertebra, acromion, anterior superior iliac spine (ASIS), and the inferior angle of the scapular, posterior superior iliac spine (PSIS), iliac crest, upper thorax, middle thorax, and lower thorax. Then, the subjects stood on the posture pad and photographs of subjects were taken in the lateral, anterior, and posterior views (Figure 1). Data of photography was transferred to the Body Style Analyzer (South Korea, LU Commerce). We analyzed FHP in the lateral view (Figure 2), and the intrarater and interrater evaluations of photogrammetry findings in the standing sagittal posture of the cervical spine were found to be reliable [26, 27]. The distance from the line through acromion to the line through the external auditory meatus was measured for FHP. FHP was calculated using the Body Style Analyzer with markings at the ear tragus and the acromion (Figure 2). If the distance was 2.5–5 cm, it was defined as moderate FHP, and if the distance was >5 cm, it was defined as severe FHP [28]. We recruited subjects with FHP >2.5 cm.

The trunk posture, including shoulder tilting, scapular level, pelvic tilting, and anterior tilting degrees in anterior, posterior, and lateral views, was obtained using the Body Style Analyzer.

**Figure 3:**
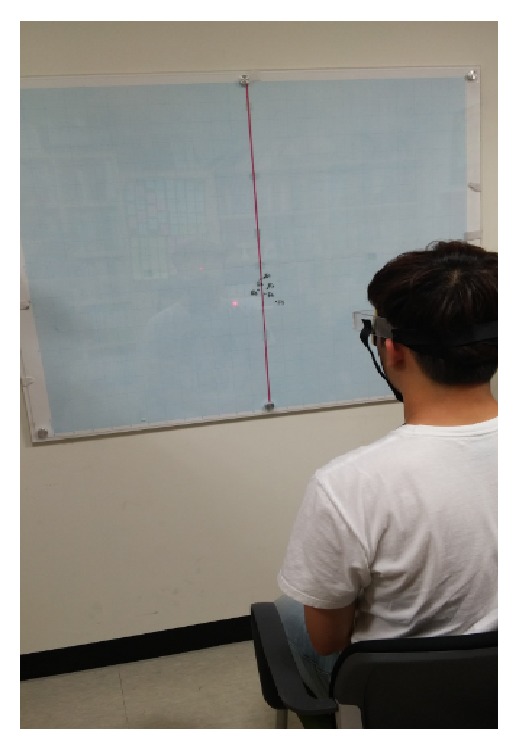
Measurement of head reposition accuracy.

For neck reposition sense testing, we used the head repositioning accuracy (HRA) test [7, 9, 29] because the head-to-neutral test has been reported to be more sensitive than the head-to-target test [30]. First, subjects were made to sit on a wooden chair with hips and knees at approximately 90° flexion and feet hip-width apart. The HRA test was performed to measure differences in measurements between the reference positions (position 0) and return positions. Equipment with a laser (Figure 3) was firmly placed on the subjects' heads. With their head in a natural resting position, the subjects were requested to focus on a target that was positioned at the eye level. All subjects were then instructed to close their eyes with a sleep shade and were instructed to memorize this position because this was the reference position. Then, they performed a full neck flexion at their preferred speed and held this position for 5 s. After this, the subjects were instructed to return to the reference position with their preferred speed. The stopping point of the laser beam was marked with a dot that was the return position.

The projection point on the abscissa and ordinate axes were measured (*X*, *Y*), and each coordinate was given a positive or negative value according to its position relative to the corresponding axis. Using these 2 values, the subject's HRA was then calculated trigonometrically. This measurement represented the direct distance between the points (the return position) on which the light beam stopped on the target to point 0 (the reference) of the target. For comparison of the absolute values for the horizontal values for the horizontal (*X*) and vertical (*Y*) components of the repositioning error, the negative signs were removed by calculating the RMS values [31]. Three repetitions of HRA to the reference position were performed and then the mean value of the trails was calculated. The same procedure was followed for extension, rotation, and side flexion, which were randomly performed (Figure 3).

### 2.3. Statistical Analysis

All statistical analyses were performed using IBM SPSS Statistics (version 20.0, IBM Corporation, South Korea). Descriptive statistics (mean and standard deviations) were calculated for each variable. Spearman's rank correlation coefficients and regression analysis were used to assess the degree of correlation between postural evaluation items and the value of each joint reposition sense, and the significance level of *α* = 0.05 was considered.

The root mean square error (RMSE) among the trials for each subject was defined by the following equations [32]:

(1) E2=x2+y2,RMSE2=1m∑i=1mEi2.

*E* denoted the differences between the initial reference position (*x*) and the final position (*y*) when repositioning from either flexed, side flexed, extended, or rotated neck position, and *m* denoted the trial number.

## 3. Results

The mean HRA values of neck flexion and extension were 9.88 and 9.68, respectively. The mean HRA values of right and left side neck flexion were 8.70 and 10.26, respectively. The mean HRA values of right and left neck rotation were 9.54 and 9.80, respectively. The mean shoulder tilting angle was 1.44 degrees, and pelvic side tilting angle was 1.52 degrees. The pelvic anterior tilting angle was 11.76 degrees, and the scapular level angle was 2.37 degrees (Table 1).

There were significant correlations between the HRA of right side neck flexion and pelvic side tilt angle (*p* < 0.05). If pelvic side tilting angle increases by 1 degree, right side neck flexion increased by 0.76 degrees (*p* = 0.026) (Table 2). However, there was no significant correlation between other HRA values including those for neck flexion, extension, right side flexion, right rotation and left rotation, and trunk posture (*p* > 0.05) (Table 3).

## 4. Discussion

FHP is affected by stress and incorrect postures. Owing to industrial development, the population of subjects with FHP has been increasing [28]. In particular, workers who use computers in their offices are likely at risk for FHP.

FHP can cause problems with proprioception of the neck muscles, and, therefore, a treatment plan is necessary for such patients. Moreover, proprioception can improve with direct treatment of neck muscle or with indirect treatment of the trunk posture, including treatment of the pelvic posture [2, 24, 33]. However, research on the indirect method is limited. In addition, further research is needed on the relationship between proprioception and trunk posture, before an indirect method can be developed.

Therefore, this study explored the correlation between HRA and trunk posture in 41 subjects with FHP to determine the relationship between proprioception of neck and trunk posture. We found a significant correlation between the right side flexion reposition sense of the neck and the pelvic side tilting angle.

Black et al. [33] found that a change in lumbar posture was associated with a compensatory change in cervical position. Murphy et al. [24] used manipulative therapy on the cervical spine to relieve low back pain. Nansel et al. [34] found that cervical spine manipulation has significant effects on the tone of the lumbopelvic musculature, particularly in the gluteal region, and Hyoung et al. [35] found that increasing cervical motion after ankle joint therapy is helpful.

Corrective exercises for FHP had a positive effect on spinal posture in patients with lumbosacral radiculopathy [36] or adolescent idiopathic scoliosis [37].

According to earlier studies, function of cervical improved after the patient received therapy for trunk and ankle region. In this study, we found similar results with those of previous studies that lumbar posture is related to cervical motion.

However, the mechanism underlying how treatment on the cervical region affects the pelvic area is unknown.

Nansel et al. [34] and Murphy et al. [24] suggested this effect of treatment may be because of its effect on the tonic neck reflex (TNR). TNR alters the tone of the trunk and extremities in two ways. One is via afferents from muscle spindles to the vestibular nucleus and the pontine and medullary reticular formation. The other is via signals from the upper cervical afferents sent to propriospinal neurons.

Therefore, if cervical dysfunction is corrected, the tone normalizes with normal patterns of the TNR, and pelvic distortion will improve.

Ahn [2] and Hyoung et al. [35] explained the treatment effect using the mechanical chain of joint. They assumed that the entire body is connected in a chain that affects each segment.

In our opinion, this relationship may be explained with two reasons: the fascia and functional structure of quadratus lumborum (QL) and scalenus muscle.

With respect to the fascia, the agonist muscles of neck side flexion and the pelvic side tilting angle are connected via the fascia on the lateral line [25].

The QL is not directly part of the lateral line according to anatomy trains' rule. However, with respect to the functional structure, the QL uniquely works as a lateral flexor of the trunk and the scalenus works as a lateral flexor of neck, similar to the QL. The QL pulls from one end of the rib cage and the scalenus from the other end. Therefore, the two muscles are very close related to functional structure. If the scalenus pulls the ribcage, it affects the QL as well, thus affecting pelvic posture. Thus, if the pelvic posture is fixed well, the proprioception of the muscles that affects movements of the neck can be refined well.

Nejati et al. [22] suggested that shoulder posture was not correlated with neck pain. However, Szeto et al. [38] found that subjects with neck and shoulder discomfort had protracted acromion posture. Lau et al. [39] specified that the sagittal posture of the thoracic spine had a very close relationship with neck pain severity and disability and suggested that thoracic posture correction would help prevent neck pain. Lynch et al. [40] found significant interactions between movement of forward head translation and those of forward shoulder translation. The results did not concur with those previously described.

The conflicting result was probably because of the lack of a gold standard in clinical measurement of posture. The methods of measurement were different in each study. Nejati et al. [22], Szeto et al. [38], and Lynch et al. [40] studied shoulder kyphosis and protraction, but Lau et al. researched the upper thoracic angle. The measurements of posture also differed among studies, indicating that there is no gold standard for the clinical measurement of posture to reflect the actual curvatures of the spine.

We could not find a correlation between shoulder posture and any neck motion, which is similar to the result of Nejati et al.'s study [22]. However, the research methodology was not the same as ours. Therefore, additional studies are needed in the future, involving the same clinical measurement. Moreover, the standard measurement of posture should be developed that can be used easily in the clinic.

Consequently, in the future, to improve the reposition sense of subjects with FHP, first, pelvic posture should be checked, and then that of its related muscles should be verified using electromyography.

The limitation of this study was that it was difficult to generalize the results owing to small sample size. In addition, in the study, we could not determine the mechanism of relationship between neck reposition sense and trunk posture. So, we will be performing an electromyography study and other equipment to confirm the mechanism in the future.

## 5. Conclusion

In conclusion, in the case of FHP subjects, the higher the pelvis side tilting angle, the worse the HRA value of lateral neck flexion. This might be the anatomical structure of the muscles around pelvic area and neck with fascia and functional structure of QL and scalenus muscle and the mechanism of that result should be studied in the future. Therefore, verifying pelvic posture should be prioritized when movement is limited due to vitiation of the proprioceptive sense of the neck owing to FHP.

## Figures and Tables

**Table 1:** The evaluation of HRA of neck motions and trunk posture.

	HRA of NF	HRA of NE	HRA of NRSF	HRA of NLSF	HRA of NRR	HRA of NLR	STA	PSTA	PAT	ScL

M ± SD	9.88 ± 5.46	9.69 ± 4.53	8.70 ± 3.99	10.26 ± 6.23	9.54 ± 3.76	9.80 ± 5.19	1.44 ± 1.05	1.52 ± 1.01	11.76 ± 6.39	2.37 ± 1.93

M: mean, SD: standard deviation, and unit: degree.

HRA: head repositioning accuracy, NF: neck flexion, NE: neck extension, NRSF: neck right side flexion, NLSF: neck left side flexion, NRR: neck right rotation, NLR: neck left rotation, STA: shoulder tilting angle, PAT: pelvic anterior tilting, PSTA: pelvic side tilting angle, and ScL: scapular level.

**Table 2:** Spearman's rank correlation coefficient between HRA of neck motions and trunk posture.

	HRA of NF	HRA of NE	HRA of NRSF	HRA of NLSF	HRA of NRR	HRA of NLR	STA	PSTA	PAT	ScL
HRA of NF	1.000	.020	−.018	.186	.118	.068	−.167	.173	.171	−.049
HRA of NE	.020	1.000	−.008	.078	.378^*∗*^	.166	.224	−.111	−.003	−.213
HRA of NRSF	−.018	−.008	1.000	.410^*∗∗*^	.016	−.035	.132	.376^*∗*^	.000	.045
HRA of NLSF	.186	.078	.410^*∗∗*^	1.000	.178	.121	.117	.243	−.188	.090
HRA of NRR	.118	.378^*∗*^	.016	.178	1.000	.290	.207	−.029	.016	−.072
HRA of NLR	.068	.166	−.035	.121	.290	1.000	−.069	−.146	−.071	−.100
STA	−.167	.224	.132	.117	.207	−.069	1.000	.157	−.185	.266
PST	.173	−.111	.376^*∗*^	.243	−.029	−.146	.157	1.000	.169	.023
PAT	.171	−.003	.000	−.188	.016	−.071	−.185	.169	1.000	−.066
SL	−.049	−.213	.045	.090	−.072	−.100	.266	.023	−.066	1.000

HRA: head repositioning accuracy, unit: degree.

NF: neck flexion, NE: neck extension, NRSF: neck right side flexion, NLSF: neck left side flexion, NRR: neck right rotation, NLR: neck left rotation, STA: shoulder tilting angle, PAT: pelvic anterior tilting, PSTA: pelvic side tilting angle, and ScL: scapular level.

^*∗*^ *p* < 0.05, ^*∗∗*^ *p* < 0.01.

**Table 3:** Regression analysis of HRA of right side flexion and pelvic side tilting angle.

Predictor variable	*B*	*β*	*T*

HRA of right side flexion	0.760	0.347	2.30^*∗*^

^*∗*^ *p* < 0.05.
